# Gender-related stigma toward individuals with a history of sexual or physical violence in childhood

**DOI:** 10.1186/s12889-024-19913-9

**Published:** 2024-09-03

**Authors:** Theresia Rechenberg, Toni Fleischer, Christian Sander, Georg Schomerus

**Affiliations:** 1 Department of Psychiatry and Psychotherapy, University of Leipzig Medical Faculty, Semmelweisstr. 10, Leipzig, 04103 Germany; 2 Department of Psychiatry and Psychotherapy, University of Leipzig Medical Center, Leipzig, Germany

**Keywords:** Child sexual abuse, Child physical abuse, Child maltreatment, Gender, Stigma, Stereotypes, Communication behaviour

## Abstract

**Background:**

Stigma is a key barrier to disclosing traumatic experiences of violence in childhood with adverse consequences for help-seeking behaviour. Disclosing behavior differs by gender and the form of violence experienced. However, there is a lack of comprehensive studies that address societal perceptions of males and females with a history of sexual or physical violence in childhood. Therefore, our aim is to focus on the impact of gender on the perception of individuals who experienced sexual or physical violence in childhood.

**Methods:**

We conducted a study on a representative sample of the German general population in terms of age and gender. Participants were randomly assigned to brief case vignettes addressing sexual or physical violence in childhood. Analyses base on a sample of *n* = 659 individuals (50.1% female). Stigma was assessed through examining respondents’ readiness to address specific traumas in conversation and respondents’ attitudes toward the individuals in the vignettes. Mann–Whitney U tests were applied to check for differences between female and male victims and survivors as well as female and male respondents.

**Results:**

Our results reveal that male victims and survivors face higher negative stereotypes (harm, unpredictability) and evoke communication barriers more often when compared to female victims and survivors, especially in male respondents. Sexual violence is associated with more distinct gender differences than physical violence.

**Conclusions:**

Findings reflect greater stigma toward male victims and survivors of sexual violence than female ones. Men had a greater tendency to stigmatize – especially toward their same-gender peers. Socially ingrained gender roles may act as a basis for different communication cultures and the notion of victim-perpetrator constellations in which males are not envisaged as victims.

## Background

Sexual and physical violence against children is an issue of global concern [64]. Childhood maltreatment can have profound and extensive negative consequences for the psychological, physical, and social development of individuals across their lifespan, such as posttraumatic stress disorder [65], substance-related disorders [36], personality disorders [31], depression and anxiety [31], suicide attempts [17], obesity [66], sexually transmitted diseases [66], or behavioral problems [24, 66]. When we refer to those affected, we support making the diversity of them visible by using the term victims and survivors since in the field of childhood violence both terms can be perceived as appropriate, but address different needs [30], [49], [57]). There is a consensus in the research literature that children tend not to disclose experiences of violence until adulthood or otherwise significantly delay disclosure [37]. On the part of victims and survivors, a lack of sexuality education [53], shame, self-blame, and anticipated stigma [32] can have an impact on the decision to remain silent or to disclose. On the part of society, negative reactions to disclosure [46, 60], child sexual abuse myths (e.g., “If a boy is sexually abused then he will be gay.” [11]), negative stereotypes (e.g., females appear to be more vulnerable than males [45]), and communication barriers [55] represent components of public stigma. Stigma is a social process, that is both enacted and felt. Broadly speaking, the components of the stigma process [35], labelling, stereotyping, separation, and discrimination describe enacted stigma that is directly experienced by those stigmatized. Stigma, however, is also felt when it is anticipated or when it is internalized as self-stigma [10, 22, 35]. Stigma has the potential to exacerbate post-traumatic symptoms [56] and remaining silent about traumatic experiences can impede help-seeking and a constructive way of coping [54, 58] and can increase the risk of continuation or repetition of violence later in life [41]. Victims and survivors of physical violence are less likely to disclose than victims and survivors of sexual violence [25, 50]. Furthermore, disclosing behavior is characterized by gender differences: Male victims and survivors disclose less often and more selectively than females [25, 40]. Alaggia [1] identified reasons for refusing or delaying disclosure at the level of felt stigma: While there were some similarities in disclosure patterns between females and males, remarkable differences emerged, with males feeling more isolated and stigmatized due to the belief that boys are rarely victimized and the fear of being identified as homosexual or becoming an abuser themselves.

Gender-related differences at the level of enacted stigma are the focus of a review by Rechenberg and Schomerus [45] which documents gender differences in the societal perception of females and males who experienced violence in childhood, likely originating in socially rooted gender roles and related expectations. The authors found that males and females differ in assigned stereotypes: While blame and lacking trust are more likely to be attributed to male victims and survivors, harm is more likely to be associated with female ones. This corresponds to stereotypical attributions such as weakness, passivity, and vulnerability for females and dominance, strength, and resistance for males, which are associated with expectations regarding the behavior of victims and survivors after experiencing violence. Rechenberg and Schomerus [45] argue that being female implies a need for protection and help due to more victim-typical attributions whereas being male is associated with higher defensive capabilities to prevent violence or to stop incipient acts of violence as a result of more perpetrator-typical attributions. These expectations may indicate that male victims and survivors are seen as more untrustworthy and more blamed because victimhood is not perceived as intended for them. This heteronormative victim-perpetrator image also impacts the notion of how to cope with trauma: As results of an internet-based experiment [59] and a qualitative study [23] reveal, male victims and survivors are expected to remain silent while female victims and survivors who disclose their experiences receive acceptance and even support. Treibel and colleagues (2008) refer to “gender-specific disclosing recommendations” in this context. The stigmatization of victims and survivors of violence in childhood seems to increase when the traumatic event departs from the expected heteronormative victim-perpetrator image [45], with violence perpetrated by individuals of the same gender as the victim and survivor being seen as particularly damaging to affected individuals. This is consistent with findings from a review by Davies and Rogers [12] which showed that sexual violence against boys perpetrated by males brings homonegative attitudes in society to light.

As a part of a study that aims to establish the prevalence of stigma toward individuals who experienced childhood trauma [55], the current work focuses on *gender-related stigma toward individuals who experienced sexual or physical violence in childhood*. Since the background of the original study was a comparison of interpersonal and accidental trauma, the term “trauma” has also been adopted in this part of the study and is used synonymously with the term “physical *or* sexual violence in childhood”. Previously, there has been a lack of studies that attempt to explore physical *and* sexual violence regarding gender-specific enacted stigma more comprehensively and as a primary outcome [45]. For this, we examined readiness of respondents from the general population to reach out to adult female and male victims and survivors of childhood trauma in conversation, as a potentially relevant specific manifestation of stigma, and the prevalence of negative stereotypes about females and males who experienced childhood maltreatment. We aim to address the following research questions:

1. To what extent does the gender of victims and survivors who experienced (sexual or physical) violence in childhood affect respondents’ readiness to address the trauma in conversation?
2. To what extent does the gender of victims and survivors who experienced violence in childhood impact societal perceptions of stereotypes such as unpredictability, harm, or blame?
  Several studies also provide insights into differences in perspectives on females and males who experienced violence in childhood based on respondent gender: Men are prone to be more rigorous than women in evaluating females and males with a history of childhood trauma, having more negative associations toward victims and survivors [2, 47]: For example, [48] found out that men judged a 14-year-old female less reliable and more culpable than women. Men also tend to support parental corporal punishment [3] and believe myths about sexual violence in childhood (e.g., “If the child enjoys the sexual contact, it is not considered an abuse.” [18]) more than women. Based on these insights, we expand the first two research questions to include the impact of respondent gender by addressing the following research question:
3. To what extent does respondent gender impact perceptions of female and male victims and survivors?

## Methods

This work is part of a study that aims to describe the prevalence and characteristics of stigma toward individuals who experienced childhood trauma. Detailed information about materials and methods can be found in the work of Schomerus and colleagues (2021).

### Sample

We conducted a population-based telephone survey among German residents aged 18 and older. Fieldwork was carried out in 2020/01 – 2020/02 by USUMA (Berlin), a leading institute specialized in market, opinion, and social research in Germany.

At the outset of the fully structured interview, respondents were presented with a short vignette depicting an encounter with a new neighbor who had gone through a traumatic experience: *“Imagine you have a new neighbor. When talking to you, they indicate that they have experienced [traumatic event] and are still dealing with the consequences.”* The vignettes had been newly constructed and pre-tested for this study (for details, see [55]).

Originally, respondents were randomly assigned one of eight versions of the scenario, differing by the gender of the neighbor and the traumatic event mentioned (sexual violence as a child (female/male) / physical violence as a child (female/male) / serious accident as a child (female/male) / physical violence as an adult (female/male)). As we focused on interpersonal trauma in childhood in the current paper, we took the scenarios contrasted in Table 1 as the basis for our analyses. Our sample thus amounts to *n* = 659 individuals.

**Table 1:** Selected scenarios of childhood trauma experiences

Female neighbor		Male neighbour	
Sexual violence as a child	Physical violence as a child	Sexual violence as a child	Physical violence as a child
*n* = 164	*n* = 165	*n* = 166	*n* = 164

As Table 2 shows, the sex distribution of the subsample was similar to that of the general population [15]. However, participants in our sample were slightly older and better educated than the general population.

**Table 2:** Socio-demographic characteristics of the study sample in comparison to the German population

	**Total population of Germany [%]**	**Sample* N* = 659 [%]**
**Sex**		
Male	48.9	49.9
Female	51.1	50.1
**Age**		
18–29	16.0	10.9
30–39	15.7	12.3
40–49	14.5	13.4
50–64	27.5	31.3
65–74	12.8	16.7
75 +	13.5	15.5
**Educational attainment**^**a**^		
< 10 y	28.5	14.3
10 y	32.1	29.3
> 10 y	35.2	55.5

Percentages of sample (*N* = 659) and reference population aged 18 + years if not otherwise specified

Population data from the Federal Statistical Office of Germany [15]

^a^Population and sample data (*N* = 659) for individuals 25 + years of age, since population data for < 25 years of age is not available

## Materials

### Readiness of addressing childhood trauma in conversations with victims and survivors

In order to learn more about how biased people are when communicating with individuals who experienced the type of violence mentioned in the vignette, participants were presented with the question “*How likely would it be that you actively raise this topic again with your neighbor?”* after receiving the vignette. Answers were given on a 5-point Likert scale ranging from 1 *very likely* to 5 *very unlikely*.

### Stereotypes

To measure beliefs about individuals with childhood trauma, we newly framed and pre-tested potential negative or positive attributions for individuals who experienced the type of trauma described in the vignette [54, 55]. For the current study, we chose the following three stereotypes as they have already been examined in several other studies [45] as common stereotypes in the perception of victims and survivors of sexual or physical violence: unpredictability (*“People who have experienced [traumatic event] are unpredictable.”*), harm (*“[…] are harmed for the rest of their lives.”*) and blame (*“[…] are guilty of what has happened to them to a certain degree.”*). Answers were given on a 5-point Likert scale, ranging from 1 *strongly agree* to 5 *strongly disagree*.

### Statistical analysis

Because we were solely interested in whether respondents would address physical or sexual violence in childhood again once their neighbor had talked about it, we reduced the 5-point Likert scale to three outcomes by combining 1 and 2 (*very likely / likely*) to *probably* and 4 and 5 (*unlikely / very unlikely*) to *probably not*. Option 3 (*indecisive*) remained as before. We applied a similar procedure to stereotypes (1 and 2 = *strongly agree / agree* summarized to *agree*, 3 = *indecisive*, 4 and 5 = *disagree / strongly disagree* summarized to *disagree*).

First, the data were stratified by the type of experienced violence (sexual violence, physical violence). A Mann–Whitney U test was applied to assess differences between female and male victims and survivors. In the second step, the data were additionally stratified by respondent gender. A Mann–Whitney U test was applied again to analyze whether respondent gender was associated with discrimination between female and male victims and survivors. Analyses were performed with “R” version 4.2–1 [44] using the package “coin” version 1.4–2 [29].

## Results

Table 3 shows the results of the Mann–Whitney U Test. Z-Scores that are further from zero denote higher differences between female and male victims and survivors regarding readiness toward addressing childhood violence and agreement with stereotypes. *P*-values refer to the distinction between the gender of victims and survivors. Overall, we found no significant differences in readiness to address childhood trauma and stereotyping of male and female victims and survivors, except in the context of sexual violence and the stereotypes of unpredictability on the part of female respondents (*p* < 0.01) and harm (*p* < 0.05) on the part of female and male respondents. However, at a closer look, we could identify subtle differences related to respondent gender regarding readiness to address sexual violence with female and male victims and survivors (female respondents: n.s., male respondents: *p* < 0.1) as well as agreement with the stereotype of harm (female respondents: n.s., male respondents: *p* < 0.1). Further results are described below.

**Table 3:** Results of the Mann-Whitney U tests: P-values showing differences in the readiness of respondents (all, female, male) to address childhood trauma if the victim and survivor was female vs. male, and to what extent respondents agree or disagree with certain stereotypes regarding female vs. male victims and survivors

		**Vignette**			
		**sexual violence**		**physical violence**	
	Respondents	Z-score	*p*	Z-score	*p*
**Readiness of addressing childhood trauma**					
	female + male	1.599	0.110	1.589	0.112
	female	0.503	0.615	1.106	0.269
	male	1.811	*0.070*	1.056	0.291
**Stereotypes**					
unpredictability					
	female + male	-1.462	0.144	-0.150	0.881
	female	-2.629	**0.009**	-0.202	0.840
	male	0.623	0.534	0.037	0.970
harm					
	female + male	-2.006	**0.045**	-1.421	0.155
	female	-0.896	0.370	-0.855	0.392
	male	-1.922	*0.055*	-1.164	0.244
blame					
	female + male	0.076	0.940	0.610	0.542
	female	0.206	0.837	0.757	0.449
	male	-0.111	0.912	0.203	0.839

### Readiness of addressing childhood trauma in conversations with victims and survivors

**Fig. 1:**
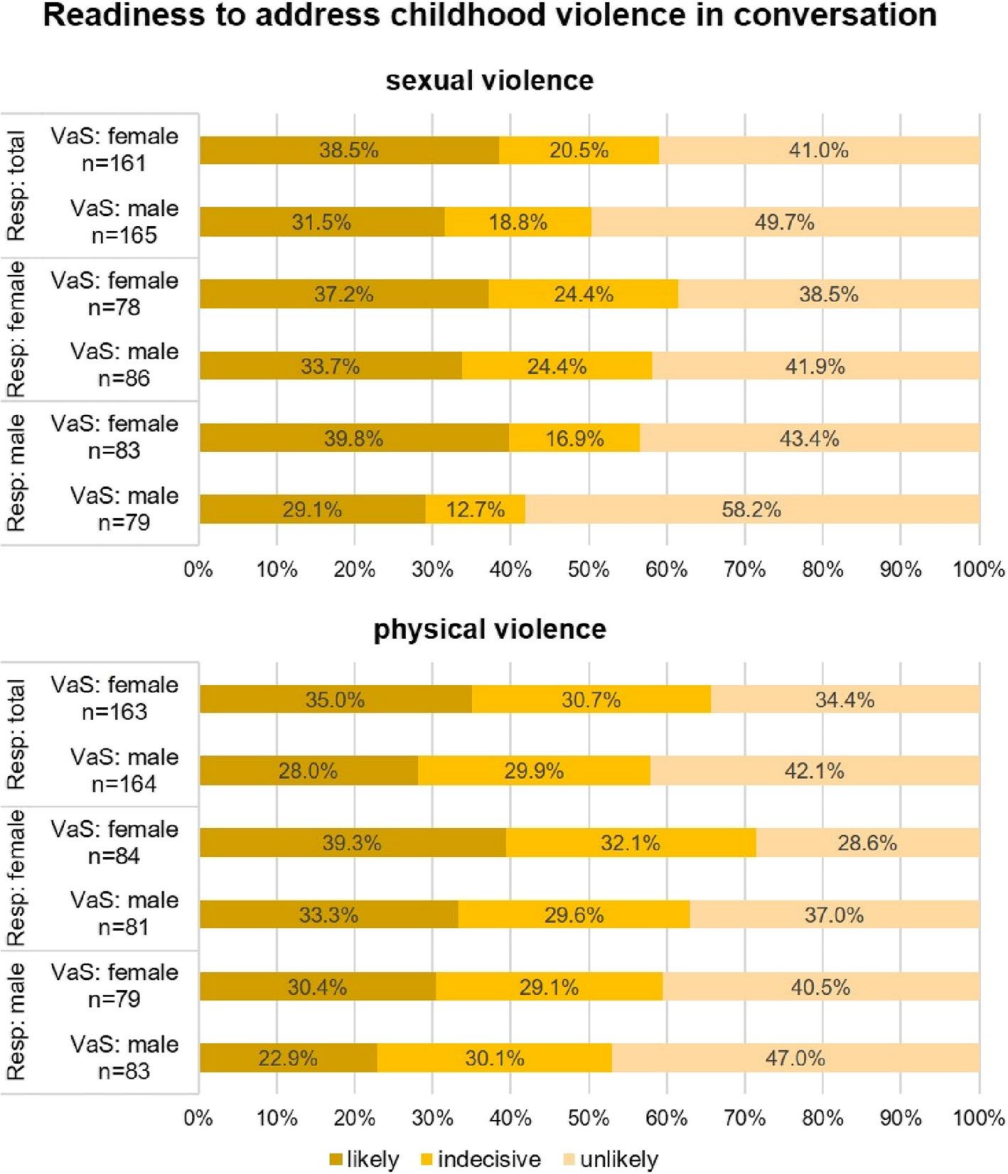
Readiness to address childhood violence in conversation (VaS = victims and survivors; Resp = respondents)

In the context of sexual violence, addressing victims and survivors tends to be avoided in general (see Fig. 1, sexual violence). However, respondents indicated that they were more likely to address trauma with female victims and survivors (38.5%) than with male ones (31.5%) while contact with male victims and survivors (49.7%) was reported to be more likely avoided than contact with female ones (41%).

Figure 1 (sexual violence) reveals that female respondents tended to show fewer differences in their contact behavior toward female and male adults who experienced sexual violence in childhood, while male respondents indicated being more likely to avoid contact with male victims and survivors than with female ones when addressing the topic of sexual violence (*p* < 0.1). Furthermore, male respondents stand out due to their much less pronounced indecisiveness and more pronounced polarization in comparison to female respondents.

When addressing physical violence, respondents showed a similar pattern as with sexual violence, but with more respondents stating that they were indecisive (see Fig. 1, physical violence): 35% of respondents would actively raise the topic again with female neighbors who experienced physical violence in childhood, whereas 28% of respondents would do so for male neighbors. 34.4% of respondents indicated to avoid contact with female victims and survivors and 42.1% of respondents stated to avoid contact with male victims and survivors. As Fig. 1 (physical violence) reveals, male respondents showed the highest reluctance, particularly concerning males with physical childhood trauma: Male respondents reported to be most likely reluctant (47%), the second most likely to be indecisive (30.1%), and the least likely to be willing (22.9%) to talk about childhood physical trauma. Male respondents also tended to avoid contact with female victims and survivors, but less rigidly than for males. Female respondents showed the reverse pattern with respect to female victims and survivors: They indicated to be most likely ready for contact (39.3%), the second most indecisive (32.1%), and the least likely to be reluctant (28.6%) to talk about childhood physical trauma.

### Stereotypes

#### Unpredictability

**Fig. 2:**
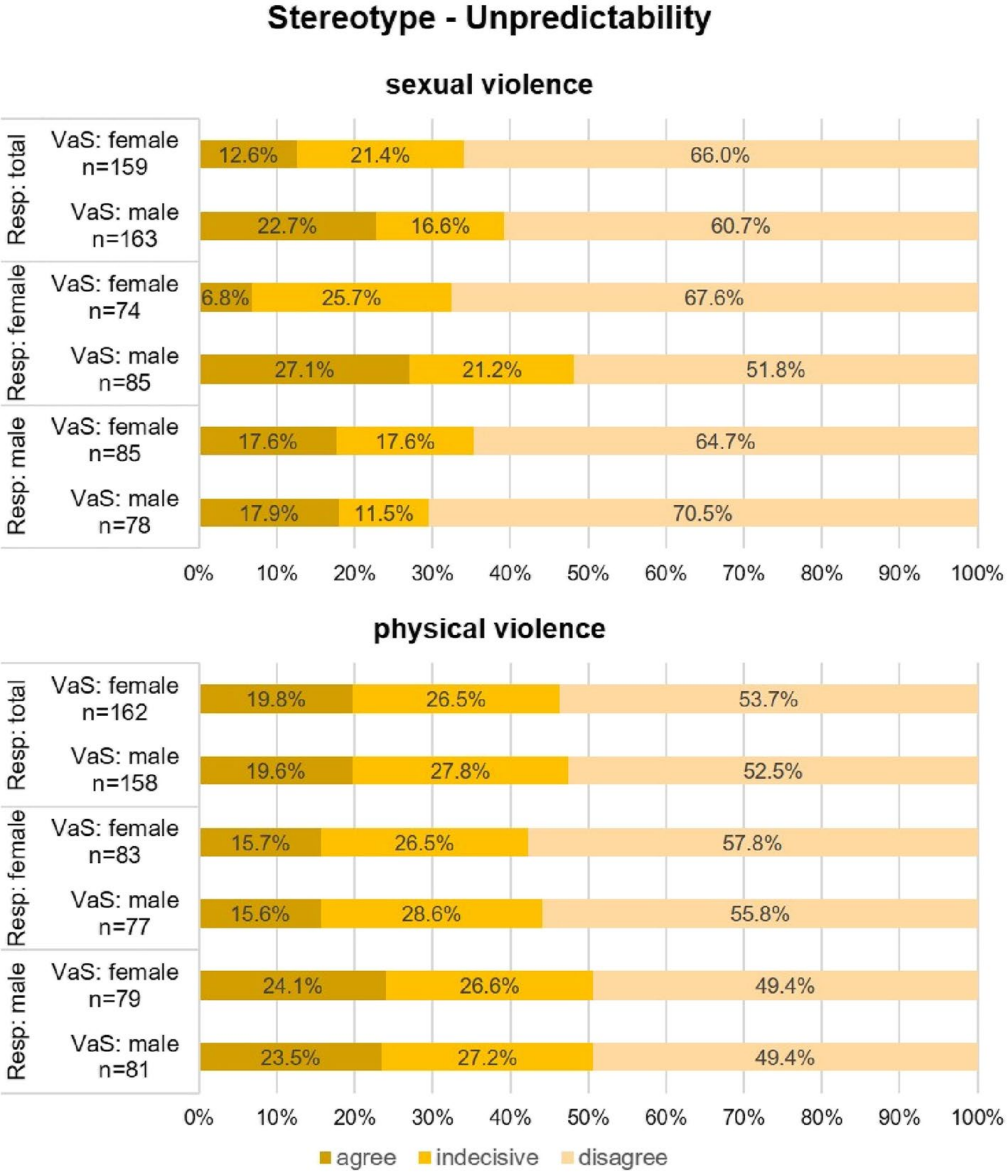
Stereotype—Unpredictability (VaS = victims and survivors; Resp = respondents)

In the context of sexual violence, the stereotype of unpredictability is rejected by a large proportion of respondents, and more often in relation to female victims and survivors (66%) than to male ones (60.7%) (see Fig. 2, sexual violence).

As can be seen in Fig. 2 (sexual violence), male respondents make only marginal distinctions between female and male victims and survivors (*p* = 0.5) when stereotyping in terms of unpredictability, whereas female respondents differentiate more rigorously (*p* < 0.01): They were four times more likely to agree with unpredictability for male victims and survivors than for female ones.

In the context of physical violence, there is only a minimal distinction between female and male victim and survivor gender when stereotyping in terms of unpredictability (see Fig. 2, physical violence). Female and male respondents show a similarly balanced picture in the attribution and rejection of unpredictability concerning female and male victims and survivors. However, female respondents are more likely to reject and less likely to assign the stereotype than male respondents (see Fig. 2, physical violence).

#### Harm

**Fig. 3:**
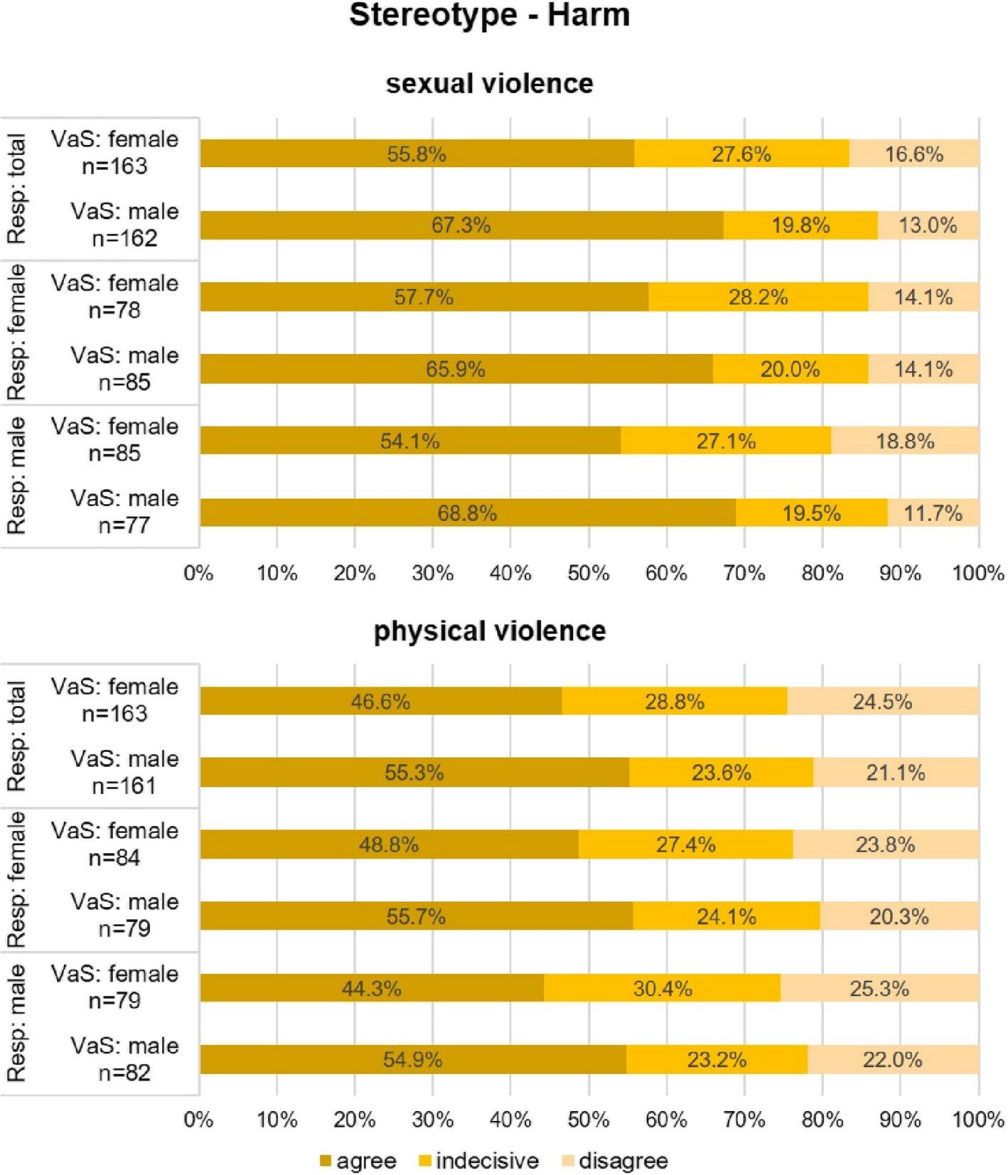
Stereotype—Harm (VaS = victims and survivors; Resp = respondents)

In the context of sexual violence, respondents largely agree with the stereotype of being harmed for the rest of their lives after an experience of violence (see Fig. 3, sexual violence), but clearly differentiate between female and male victims, which is a significant finding (*p* < 0.05): While 55.8% of respondents consider female victims and survivors to be harmed, 67.3% of respondents agree with the stereotype for male victims and survivors.

Figure 3 (sexual violence) shows that both female and male respondents have a similar response pattern with respect to gender differences in the attribution of harm, which was more pronounced in male respondents.

In the context of physical violence, a similar response pattern emerged among respondents as in the context of sexual violence (see Fig. 3, physical violence): A clear majority of respondents shows agreement with the stereotype of being harmed for the rest of the life after an experience of violence for female (46.6%) and male (55.3%) victims and survivors. The response pattern of female and male respondents remains similar to that for sexual violence although ratings of agreement with harm for both female (48.8% and 44.3%, resp.) and male (55.7% and 54.9%, resp.) victims and survivors decline (see Fig. 3, physical violence).

#### Blame

**Fig. 4:**
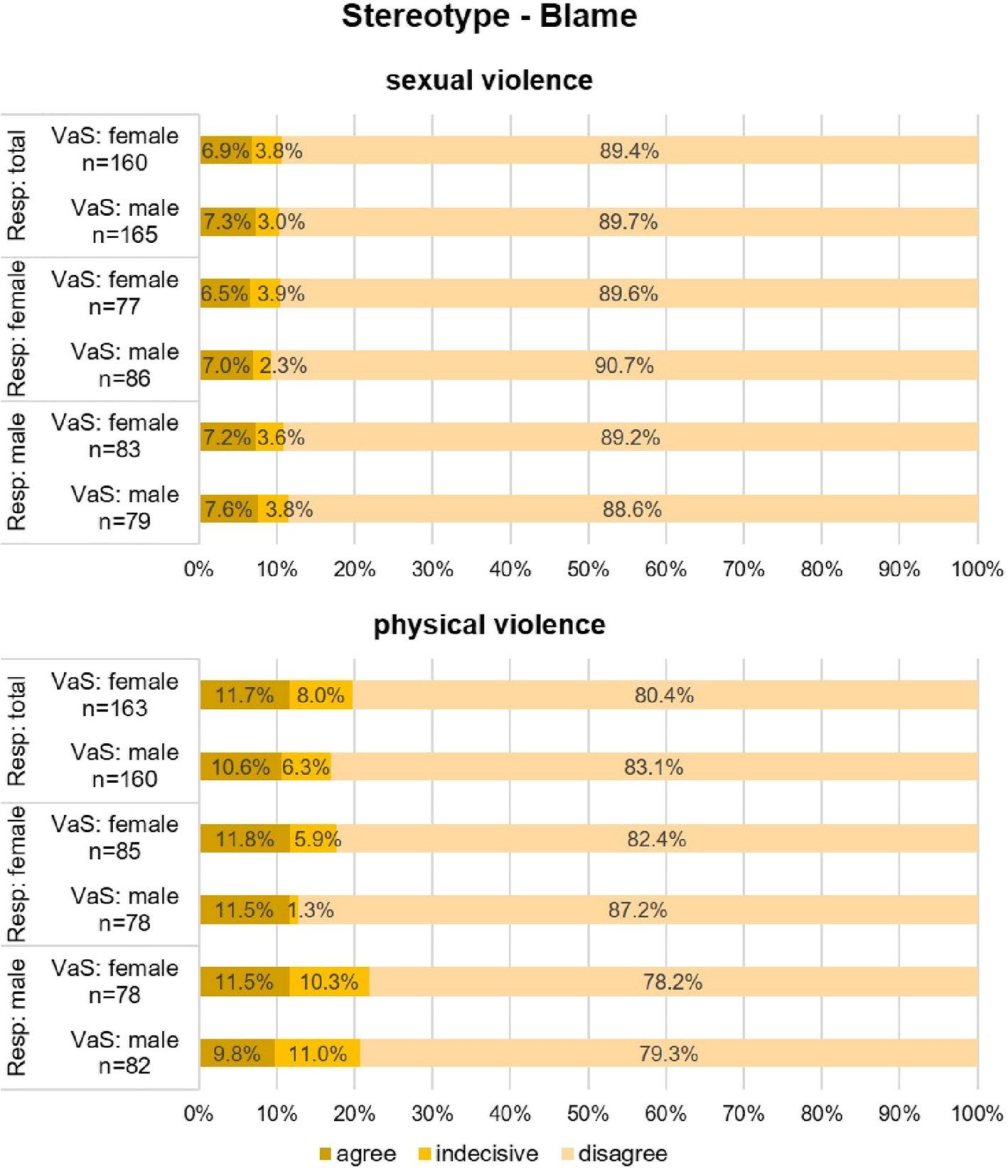
Stereotype—Blame (VaS = victims and survivors; Resp = respondents)

In the context of sexual violence, the stereotype of being blamed is rejected by a substantial and almost identical proportion of respondents for both female (89.4%) and male (89.7%) victims and survivors (see Fig. 4, sexual violence). There are very subtle differences between response rates of female and male respondents. However, male respondents tend to agree with the stereotype of blame more than female respondents (see Fig. 4, sexual violence).

In the context of physical violence, the picture is similar, but shifts slightly toward being indecisive or agreeing with the stereotype of blame (see Fig. 4, physical violence). The responses of female and male respondents show a slightly more heterogeneous picture in agreeing or disagreeing with the stereotype of blame toward female and male victims and survivors in the context of physical violence (see Fig. 4, physical violence). Female respondents generally disagree more with the stereotype of blame and are less likely to be indecisive than male respondents. Male victims and survivors are least likely to be considered blamable by both female (disagree: 87.2%, indecisive: 1.3%) and male (disagree: 79.3%, indecisive: 11.0%) respondents.

## Discussion

This work examined to what extent the gender of victims and survivors who experienced sexual or physical violence in childhood impacts (1) readiness to address the trauma in conversation and (2) the societal perceptions of stereotypes such as unpredictability, harm, or blame. In addition, (3) we expected the inclusion of the respondent gender to concretize the findings.

### Readiness of addressing childhood trauma in conversations with victims and survivors

Our results reveal a pattern of greater reluctance to address male victims and survivors of childhood trauma compared to female ones, particularly regarding sexual violence. This pattern is more pronounced in male respondents, who are the most reluctant to address male victims and survivors, while female respondents appear less rigorous in their response behavior and more often indicate being indecisive.

The greater tendency to avoid contact with victims and survivors of sexual violence compared to those who experienced physical violence can potentially be derived from a sociocultural perspective: While sexual violence is taboo and clearly ostracized, experiencing physical violence in childhood in the form of ‘educational measures’ is socially accepted to a certain extent, with corporal punishment not being prohibited by law in many parts of the world [21]. While corporal punishment in educational institutions had been banned in the German Democratic Republic since its founding in 1949 [20], it was permitted in the Federal Republic of Germany. In 2000, a decade after the reunification of Germany, a child’s right to a violence-free education and upbringing was enshrined in law. This amendment entails attitude changes toward the use of violence against children revealing a decrease in the endorsement of corporal punishment in education [43]. However, a recent survey of the German general population revealed that milder forms of physical violence (e.g., a bottom smack) are still accepted in society, with men condoning physical violence more than women [8].

The taboo of sexual violence is reinforced by the inconsistency of masculine ideals – being strong, dominant, and capable of defending themselves – and the image of a victim, especially in the context of sexual violence: In a review, Rechenberg and Schomerus [45] show that a deviation from expected heteronormative victim-perpetrator constellations that does not envisage boys as victims and involves the idea of a male perpetrator is associated with stronger stigma, which manifests in the attitudes of society [5, 6] as well as in the anticipations of stigma in victims and survivors [1, 27]. The homosexual character of sexual violence against boys might be accompanied by feelings ranging from insecurity to discomfort to averseness toward male victims and survivors – especially on the part of men, since men tend to be significantly more homonegative than women [34, 39]. These feelings might result in the avoidance of conversations with male victims and survivors. Conversely, there could be a greater willingness to address trauma with females to express sympathy and support for more stereotypical victims.

Another explanation for the findings may be that females and males have internalized different communication cultures. As women are associated with more social, emotional, and forthcoming way of communicating than men [38], the threshold to addressing childhood violence in conversation might be lower both toward female victims and survivors and on the part of female respondents. Mental health issues seem to exacerbate these communication differences: Basow and Rubenfeld [4] state that „men are less encouraged and women more encouraged to develop traits that enhance interpersonal problem-based communication” [4], p. 187). Consequently, if men assume that talking about traumatic experiences, which automatically indicates unmanly vulnerability, opens up another level of weakness, it is conceivable that men draw conclusions from themselves to others and therefore tend to avoid contact with male victims and survivors. Moreover, even if a problem is identified, there is a lack of vocabulary and knowledge to address it and to identify ways in which male victims and survivors can get the help they need [14]. Treibel and colleagues (2008) show that society reflects this stereotypical communication behavior in gender-specific disclosure recommendations: Females who are perceived as typical victims are expected to share and be helped whereas males are recommended to remain silent.

### Stereotypes

Overall, when stereotypes are attributed, male victims and survivors are more likely to be addressees than female ones. There is slightly more uncertainty in assigning stereotypes to female victims and survivors than to male ones.

### Unpredictability

Respondents are more likely to reject the stereotype of unpredictability than to agree with it. While there is hardly any difference in attitudes toward female and male victims and survivors who experienced physical violence, male victims who experienced sexual violence are more often associated with unpredictability than female ones, especially by female respondents. Female respondents are more rigorous in their agreement and disagreement with the stereotype in favor of female victims and survivors, revealing the perception that males who experienced sexual violence in childhood are likely to be a potential threat. The myth that males who were abused as boys are likely to become perpetrators themselves is well-known [18] and has been broadly discussed in the public media, potentially influencing attitudes of females accordingly. Regardless, sexual violence appears to be a far more sensitive topic, with respondents in the present study showing more polarized response behavior and a greater disagreement with the stereotype when compared to physical violence.

### Harm

The stereotype of being harmed for the rest of one’s life is assigned primarily to male victims and survivors and is applied more to individuals who experienced sexual violence than physical violence. At first, this result seems to contradict stereotypical gender attributes such as vulnerability and weakness for females, by which female victims and survivors could be seen as more likely to be harmed after experiencing violence in childhood [45]. Our results suggest a different conclusion: Precisely *because* males are considered dominant, strong, and capable of defending themselves, violence experienced against all expectations could lead to them being perceived as flawed and deficient in their potential to live up to the male role model, which in of itself constitutes a form of harm. This explanation is supported by the fact that male respondents make greater distinctions between female and male victims and survivors than female respondents and are most likely to agree with the stereotype toward male victims and survivors. As male respondents carry the same social expectations, they might have a stronger sense of concern and thus a more decisive image of harm than female respondents.

Associations with homosexual violence are likely to increase perceived harm for male victims and survivors. Despite the continuous decline of classical homonegativity in Western cultures, such as granting of equal rights, a more subtle form of homonegativity is still present [34]. Homosexuality is still considered at least implicitly a threat to masculinity [27]. Studies revealed that men show higher levels of homonegativity compared to women [19, 51], and that males have more negative attitudes toward gay men than toward lesbians [51], which might explain why male respondents in this study hold more negative stereotypes toward males who experienced sexual violence in childhood than toward females. Male victims and survivors mirror this homonegativity by viewing „homosexuality […] as an evitable harm transmitted by sexual victimization “ [27], p. 11).

### Blame

A considerable number of respondents disagree with the stereotype of blame in the context of violence in childhood. Victims and survivors of sexual violence evoke a slightly higher rejection of blame than those who experienced physical violence. Respondents make very few distinctions between the genders of victims and survivors, with female victims and survivors of physical violence and male victims and survivors of sexual violence being most likely to be blamed for their own abuse.

Several studies have revealed that male victims and survivors are blamed more often for sexual and physical violence than females [45]. Against this backdrop, our finding that blame is generally given little importance in the perception of victims and survivors and that their gender is no reason to differentiate appears to be unexpected. However, upon closer examination, two explanatory approaches can be identified: (1) Because blame is a relatively severe allegation toward victims and survivors in the context of childhood violence and addresses accountability in direct temporal relation to the violent act(s), it is conceivable that little connection is seen between violence in childhood and blame,(2) the topic of violence in childhood has received increased attention as a result of various media campaigns [13] [61], [62] and reporting, e.g., on police crime statistics [9], which raises societal awareness of facts and context factors related to childhood violence, thus counteracting the stereotype of blame.

### Limitations

The findings of the present study must be interpreted in light of several limitations. Since we conducted interviews by telephone calls, the answers of the study participants may be biased in terms of social desirability [28]. Whilst we collected data on the respondent’s sex and age, we did not collect data on their race, ethnic, or religious background, although these characteristics may have had an impact on our outcome. The exploratory character of the study results in a rather small sample, which limits the representativeness of the results. Current social developments may have had an influence on respondents’ answering behavior: During the period of the survey, the issue of “child sexual abuse within the Catholic Church” was very present in the media. With the knowledge that mainly boys were affected by sexual violence in this context [16], the victimhood of males may have lost its scarcity value and been more normalized. As a consequence, respondents might have positioned themselves less rigorously.

With regard to the vignette scenario, respondents were left room for interpretation, which may have led to a bias in responses: (1) It remains uncertain what associations respondents have regarding the neighbor’s nature and the age of the victim at the time of experienced violence, as previous individual experiences with neighbors can be varied. According to the Gender Intensification Hypothesis [26], gender role expectations increase with the age of children, entailing a varying level of stereotyping depending on what age was associated with childhood trauma. (2) Furthermore, neighborly relationships differ in terms of actual or perceived closeness and distance, and culturally and individually determined ways of behaving such as discretion, courtesy, or sense of tact. (3) It is necessary to note that although the violence addressed in the vignette was experienced in childhood, the encounter with the person affected occurs in adulthood. Consequently, respondents’ perceptions and evaluations could be biased due to the age discrepancy.

On the part of researchers, there is also room for interpretation, which primarily concerns the question about addressing childhood trauma: The respondent’s motives regarding avoiding or seeking contact with victims and survivors who disclosed their trauma experiences remain unknown. The finding that male victims and survivors of sexual violence are approached less frequently, concluding that they are not perceived as typical victims may be based on different motives: Uncertainty in handling an unusual situation, consideration for victims and survivors (e.g., not wanting to insult someone as a homosexual), or aversion, especially on the part of male respondents, since male victims and survivors do not conform to the image of masculinity and additionally undermine masculinity through a potential homosexual character of violence.

## Conclusions

Our study is, to our knowledge, the first to address gender-specific enacted stigma of individuals who experienced physical or sexual violence in childhood more comprehensively and as a primary outcome. The results emphasize that an analysis with a particular focus on gender proves beneficial, as not only the form of violence, but also the gender of victims and survivors and respondents impacts the perceptions of individuals with a history of sexual or physical violence in childhood. Despite the limitations, we were able to show significant gender effects and patterns in response behavior, pointing to male victims and survivors of childhood trauma being the focus of negative stereotypes more often and evoking communication barriers more often than female victims and survivors: Overall, we found a greater stigma toward male victims and survivors of sexual violence than female ones. There were similar, but less distinct results concerning physical violence. The predominantly more rigorous response behavior of male respondents indicates that men have a greater tendency to stigmatize – especially toward their same-gender peers.

Our findings suggest that socially ingrained gender roles may act as a basis for the perception of a victim-perpetrator constellation and the concomitant atypical victimhood of males, as well as the potentially complicating homosexual character of the experienced sexual violence. We assume that liberalization tendencies, with regard to gender justice on the one hand and a heightened awareness of the extent of violence in childhood on the other, are emerging in the German population and contribute to mitigating gender effects.

### Implications for practice and future research

It remains a political and societal obligation to continue raising awareness of violence in childhood, to critically question gender stereotypes in education, and to train educators, in particular, to recognize possible experiences of violence in both girls and boys and offer adequate help.

Future research should address not only gender-specific perceptions of individuals with childhood trauma but also identify barriers and suitable pathways to help-seeking regarding gender in both victim and survivor roles. As significant effects of emotional maltreatment and neglect in childhood are described in the literature [42, 52], further research efforts on the perception of individuals with past traumatic experiences should expand the concept of violence to also include psychological violence and neglect. If research on experiences of violence in childhood focuses on the gender of victims and survivors, the inclusion of persons who identify as trans or non-binary might prove relevant in terms of diversity considerations, as they are at increased risk of experiencing violence in youth [7] and already belong to a highly stigmatized group [33, 63].

## Data Availability

The datasets used and analyzed during the current study are available from the corresponding author on reasonable request.
